# Catalase Activity in Keratinocytes, Stratum Corneum, and Defatted Algae Biomass as a Potential Skin Care Ingredient

**DOI:** 10.3390/biomedicines9121868

**Published:** 2021-12-09

**Authors:** Michal Szczepanczyk, Tautgirdas Ruzgas, Fredrika Gullfot, Anna Gustafsson, Sebastian Björklund

**Affiliations:** 1 Department of Biomedical Science, Malmö University, 214 32 Malmö, Sweden; michal.szczepanczyk@mau.se (M.S.); tautgirdas.ruzgas@mau.se (T.R.); anna.gustafsson@mau.se (A.G.); 2 Biofilms-Research Center for Biointerfaces, Malmö University, 214 32 Malmö, Sweden; 3 Simris Alg AB, 276 50 Hammenhög, Sweden; gullfot@hey.com

**Keywords:** antioxidants, skin care, oxidative stress, skin organ, oxygen electrode, Clark electrode

## Abstract

The generation of reactive oxygen species presents a destructive challenge for the skin organ and there is a clear need to advance skin care formulations aiming at alleviating oxidative stress. The aim of this work was to characterize the activity of the antioxidative enzyme catalase in keratinocytes and in the skin barrier (i.e., the stratum corneum). Further, the goal was to compare the activity levels with the corresponding catalase activity found in defatted algae biomass, which may serve as a source of antioxidative enzymes, as well as other beneficial algae-derived molecules, to be employed in skin care products. For this, an oxygen electrode-based method was employed to determine the catalase activity and the apparent kinetic parameters for purified catalase, as well as catalase naturally present in HaCaT keratinocytes, excised stratum corneum samples collected from pig ears with various amounts of melanin, and defatted algae biomass from the diatom *Phaeodactylum tricornutum*. Taken together, this work illustrates the versatility of the oxygen electrode-based method for characterizing catalase function in samples with a high degree of complexity and enables the assessment of sample treatment protocols and comparisons between different biological systems related to the skin organ or algae-derived materials as a potential source of skin care ingredients for combating oxidative stress.

## 1. Introduction

Reactive oxygen species (ROS) constitute a collection of molecules that are continuously generated, transformed, and consumed in all living organisms as a consequence of the fact that aerobic life relies on oxygen metabolism. ROS are traditionally viewed as oxidative stressors, which cause damage that leads to a decline of tissue and organ systems in aging and disease [1]. In addition, ROS can be induced by several other biological processes, such as cytokine-based immune responses and chronic psychological stress, as well as from exogenous sources, such as ionizing radiation, UV light, environmental pollutants, and various xenobiotics [2,3]. Uncontrolled oxidative stress due to elevated concentrations of ROS in the biological milieu represents a hazardous state that may lead to detrimental effects on a wide range of biological processes, mainly due to ROS-induced modifications of RNA, DNA, proteins, or lipids on a molecular level [1]. Here, our skin is one of the most exposed organs to oxidative stress due to the fact that it is in direct contact with the oxygen-rich external environment, in combination to exposure to UV radiation, environmental pollution, and overall, rather harsh conditions.

It is clear that increased free radical action can overwhelm the antioxidative defense mechanisms of the skin organ and intensify skin aging via pigmentation, wrinkling, and dryness, as well as the promotion of more severe skin disorders, including dermatitis and skin cancer [3,4]. To combat these issues, one main goal of many skin care products is to neutralize ROS overproduction and thereby delay the onset of skin aging by slowing down any processes leading to structural and visual skin aging effects over time. The cosmetics industry has focused on bioactive substances derived from natural products such as plants, microbial metabolites, and marine algae to meet the increasing demand of consumers for natural and sustainable sources for beneficial skin care ingredients [5,6]. In particular, the fact that many marine organisms live in complex habitats associated with extreme conditions implies that these organisms have adopted themselves to these environments by producing a wide variety of antioxidative enzymes and antioxidative metabolites that may not be found in other organisms [7,8]. Considering the well-developed antioxidative system of these organisms and the environmentally friendly process of producing them, cosmetic products based on algae biomass have gained attention both from industry as well as academia [9].

However, at present, there is a lack of in vitro methods allowing for the investigation of ROS reactions at controlled conditions where the antioxidative enzyme is residing in a realistic biological matrix, such as the skin organ or various biomaterials. The importance of antioxidative enzymes in the skin organ, such as catalase, superoxide dismutase, glutathione peroxidase, glutathione reductase, peroxiredoxin, and heme oxygenase, is emphasized by their high expression in this tissue [10]. In particular, the activity of catalase was reported to be 720% higher in human epidermis as compared to the underlying dermis [11]. Further, it has been shown that active catalase is present in the outermost layers of the epidermis [12,13,14,15]. This a remarkable finding considering that the solid-like matrix of the corneocytes or the extracellular lipids of the stratum corneum (SC) membrane [16,17,18,19,20] clearly represents a very different environment in comparison to, for example, the environment of the peroxisomes where catalase is normally found [21]. Further, reduced expression of catalase in the skin has been associated with skin diseases, such as vitiligo, polymorphic light eruption, and xeroderma pigmentosum [13,22,23]. For some skin diseases associated with reduced catalase expression, the topical application of exogenous catalases was suggested to compensate for this loss [23]. Moreover, the potential for topical formulations containing catalase, encapsulated in vesicles, was investigated with the aim of reducing oxidative stress during wound healing [24]. Nevertheless, the clinical use of antioxidant-related therapies, such as formulations containing catalase, is still relatively unexplored.

Since the discovery of catalases in 1947, this group of enzymes has been recognized as one of the most important players of the biological antioxidative system [21]. Considering this, the aim of this work is to characterize the function of this antioxidative enzyme in various biological samples related to the skin organ or potential sources of catalase to be used in topical skin care formulations. In particular, we investigated the potential of using defatted algae biomass (DAB) as a source of catalase. The DAB used in this work is a byproduct derived following the lipid extraction of the microalga *Phaeodactylum tricornutum*, which is a diatom with rapid growth, high lipid content, especially omega-3 long chain polyunsaturated fatty acids, and therefore exhibits a commercial potential as a food supplement [25]. However, in order to increase the economic feasibility of using the oleoresin fraction of this microalga as an ingredient in food supplements, it is advantageous to add value to the byproduct derived after lipid extraction, for example, by employing DAB as a source of beneficial skin care ingredients. As such, it has been shown that *Phaeodactylum tricornutum* contains a multitude of different components, in addition to its oleoresin fraction, such as polyphenols (e.g., gallic acid and rosmarinic acid) and carotenoids (e.g., fucoxanthin [26,27]). Yet, the potential of using DAB as a source of the antioxidative enzyme catalase for skin care products remains an unexplored issue, which was the reason for including this biomaterial in this study, particularly in comparison to the catalase activity in SC samples.

The main reaction of catalase is the conversion of H_2_O_2_ into H_2_O and O_2_ according to Equation (1), which can be viewed as a detoxification process. In fact, catalase is one of the most efficient known enzymes, with an impressive turnover rate of 10 million H_2_O_2_ molecules per second, and it was even proposed that it cannot be saturated by H_2_O_2_ at any concentration [28]. However, it should be noted that the catalase reaction is more complex as it occurs in two stages. During the first stage, the enzyme binds to H_2_O_2_ and breaks it into H_2_O and an O atom, which is instantaneously attached to the iron atom in a heme group. In the second stage, catalase decomposes another H_2_O_2_ molecule, which leads to the release of H_2_O and gaseous O_2_ [29]. Catalase can also take part in peroxidase-type reactions by oxidizing suitable hydrogen donors, such as polyphenols or alcohols (e.g., ethanol), with the production of aldehydes (e.g., acetaldehyde), according to Equation (2) [30].

(1) $$H_{2}O_{2}+H_{2}O_{2}\overset{\mathrm{Catalase}}{\to }2H_{2}O+{\;O}_{2},$$

(2) $$H_{2}O_{2}+{\mathrm{CH}}_{3}{\mathrm{CH}}_{2}\mathrm{OH}\;\overset{\mathrm{Catalase}}{\to }2H_{2}O+{\;\mathrm{CH}}_{3}\mathrm{CHO}.$$

Here, it may be noted that catalase is the only enzyme of the antioxidative system that produces O_2_ after exposure to H_2_O_2_. This unique feature can be taken advantage of by the oxygen electrode as a general method to determine the catalase activity in different biological materials exposed to oxidative stress from H_2_O_2_. Thus, the aim of this work is to evaluate an oxygen electrode-based chronoamperometric method for characterizing the function of catalase in various biological samples related to the skin organ or potential green sources of catalase to be used as a skin care ingredient. The presented method is based on the Clark oxygen electrode, which was employed to determine the concentration of oxygen produced by the catalase reaction, see Equation (1). Although the use of the oxygen electrode in this context was first introduced in 1967 [31], the technique has not been fully explored. In contrast to the often employed spectrophotometric methods [32,33], the oxygen electrode method allows the investigation of catalase function in biological samples in the form of suspensions, or even in the form of whole parts of excised tissue, as recently demonstrated with the skin-covered oxygen electrode [14,15]. In other words, the present method provides a more realistic representation of the enzyme behavior in its native biological environment as it avoids, for example, some parts of the biological matrix being lost during centrifugation or filtration, which is a frequent problem for optical methods in the production of clear and transparent solutions.

In summary, the purpose of this work is to assess the catalase activity and the apparent kinetics of catalase in keratinocytes and SC samples and to make a comparison with the corresponding catalase function in DAB, which may serve as a potential source of catalase and other antioxidative enzymes or metabolites to be used in skin care applications. In conclusion, the results of this work illustrate the advantages of the simple and highly available oxygen electrode method, such as its versatility and ability to provide fast and accurate measurements with small sample amounts of various biological samples.

## 2. Materials and Methods

### 2.1. Materials

Catalase from bovine liver (CAS no. 9001-05-2, MW_Tetramer_ ≈ 250 kDa, 2000–5000 U/mg), hydrogen peroxide (H_2_O_2_, 35%), tablets for preparing phosphate buffered saline (PBS, 10 mM phosphate buffer, 2.7 mM potassium chloride, and 137 mM sodium chloride, pH 7.5), sodium azide (NaN_3_), sodium hydroxide (NaOH), hydrochloric acid (HCl), Triton™ X100, and sodium dodecyl sulphate (SDS) were purchased from Sigma-Aldrich (Stockholm, Sweden). All solutions were prepared from Milli-Q water.

### 2.2. Preparation of HaCaT Keratinocytes

Human immortalized epidermal keratinocyte cell line (HaCaT, CLS Cell Lines Service GmbH, Eppelheim, Germany) was maintained in Dulbecco’s Modified Eagle Medium (DMEM) growth medium, supplemented with 4.5 g/L glucose, L-glutamine, 10% fetal bovine serum, and 1% penicillin-streptomycin from Gibco (Thermo Fischer Scientific, Waltham, MA, USA). Cells were cultured at 37 °C, 5% CO_2_ in a humidified atmosphere and were passaged with accutase cell detachment solution (Thermo Fisher Scientific, Waltham, MA, USA) at 90% confluency. For the experiments, 2 × 10^5^ HaCaT cells diluted in PBS were used.

### 2.3. Preparation of Stratum Corneum (SC)

Pig ears with clear variations of melanin content (i.e., skin color) were obtained from a local abattoir (Strömbecks, Brösarp, Sweden). Eleven pig ears with darkly pigmented skin and 17 pig ears with lightly pigmented skin were used to prepare four pooled SC batches of dark or white SC from either the backside or the inside of the ears. First, the skin tissue was dermatomed and placed on filter paper soaked in PBS, containing 0.2 wt% trypsin, at 4 °C for 12 h. Next, sheets of SC were removed with forceps and rubbed with cotton-tipped applicators to remove tissue not belonging to SC, and further washed in PBS solution. After drying the SC sheets in a vacuum desiccator for two days, the sheets were pulverized into a flaky powder with the use of a mortar and pestle. It should be noted that each of the four pooled batches contained SC tissue from all available ears; i.e., black inside (*n* = 11), black backside (*n* = 11), white inside (*n* = 17), and white backside (*n* = 17). Further, the batches were handled separately to avoid mixing between the samples to enable a comparison of the catalase activity of the different SC batches. The measurements were performed within a few days after preparing the SC samples.

### 2.4. Preparation of Defatted Algae Biomass (DAB)

DAB was obtained from Simris Alg AB, Sweden. The microalgae *Phaeodactylum tricornutum* were grown under natural sunlight and LED light in multiple bioreactors. After harvest by centrifugation, the partly dewatered biomass was ground in a bead mill, freeze-dried, and treated by supercritical carbon dioxide extraction to separate the lipophilic fraction from the DAB. The residual DAB was stored in a freezer (−20 °C) for no longer than 4 years.

### 2.5. Protocols for Sample Treatments

The various biological samples were added to a reaction vessel in the form of dispersions in PBS containing either 3.0 µg of neat catalase from bovine, 2 × 10^5^ HaCaT keratinocytes, 1.0 mg of SC, or 1.0 mg of DAB. Here, it can be noted that the SC and DAB samples were in a form of dried powder and in order to disperse these samples they were mildly sonicated. In brief, the sample dispersions were exposed to 40 s in 10 s intervals of sonication at 20 kHz and 400 W (Digital Sonifier^®^, Model S-450D, Branson, Emerson Electric Co, St. Louis, MO, USA). In this manner, a fine dispersion was obtained, which allowed for the precise addition of 1.0 mg of SC or DAB to the reaction vessel by pipetting. In addition, to disrupt the keratinocyte cell membrane and release intracellular catalase, different methods were employed, including incubation in surfactant solutions for 60 s (SDS or Triton X100) and sonication according to the protocol described above.

### 2.6. Preparation of the Oxygen Electrode

The oxygen electrode was purchased from Optronika UAB (Vilnius, Lithuania) and consisted of a 250 μm diameter platinum (Pt) electrode melted in glass with an internal Ag/AgCl reference electrode. The surface of the Pt cathode of the oxygen electrode was polished using an alumina suspension (1 µm alumina particles, Buehler, Lake Bluff, IL, USA) and rinsed with water. The body of the electrode was filled with saturated KCl solution and covered with a 5 µm Teflon membrane (Optronika UAB, Vilnius, Lithuania) before measurements.

### 2.7. Measurements of Catalase Activity by the Oxygen Electrode-Based Method

**Figure 1:**
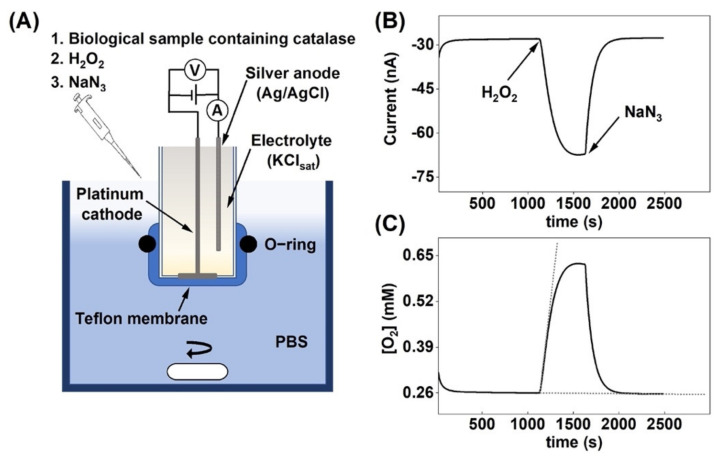
(**A**) Schematic illustration of the oxygen electrode set-up and the experimental protocol used to measure the change in oxygen concentration due to H_2_O_2_ decomposition by catalase. The electrode is immersed in glass reaction vessel filled with 5 mL of PBS, which is kept under continuous stirring. (**B**) Current response due to oxygen production after H_2_O_2_ addition followed by catalase inhibition by sodium azide. (**C**) Change of the O_2_ concentration in the reaction vessel, corresponding to the current response in (**B**). The dashed lines illustrate the tangents used to determine the initial reaction velocity $d[O_{2}]/dt$, see Equation (3).

The oxygen electrode was immersed into an electrochemical cell filled with 5 mL of PBS (see Figure 1A). In the case of measurements in alkaline or acidic environments, NaOH or HCl was used to adjust the pH of the PBS solution between 5.0 and 9.0. The current of the electrode was recorded using a CompactStat potentiostat from IVIUM Technologies (Eindhoven, The Netherlands). The oxygen electrode was connected to the potentiostat in a two-electrode configuration and the chronoamperometric measurement was conducted by applying −0.7 V vs. Ag/AgCl/KCl (sat) on the Pt cathode of the oxygen electrode. A defined amount of the sample was dispersed in PBS and added to the electrochemical cell. Here, it may be noted that the total masses of the different samples (per 5 mL of PBS) were 3.0 µg of neat catalase, 2 × 10^5^ of keratinocytes, 1.0 mg of DAB, and 1.0 mg of SC. Once a stable baseline current was established, a defined amount of H_2_O_2_ was pipetted into the electrochemical cell to achieve a specific H_2_O_2_ concentration. In the presence of active catalase, a reduction in the current of the oxygen electrode was observed due to O_2_ production, see Equation (1). In all experiments, the solution surrounding the oxygen electrode was continuously mixed with a magnetic stirrer at 400 rpm. All measurements were conducted at room temperature (i.e., 22 °C).

### 2.8. Determination of Catalase Activity

The general aim of this paper was to the evaluate the versatility of an electrochemical chronoamperometric method for measuring the catalase activity in biological systems with varying complexity. The chronoamperometric data (Figure 1B) were recalculated to obtain values in O_2_ concentration as a function of time (Figure 1C), given that the equilibrium O_2_ concentration in PBS is equal to 0.26 mM [34]. To ensure that the observed response was strictly related to O_2_ production by catalase due to decomposition of H_2_O_2_, the catalase inhibitor sodium azide (NaN_3_) was added to the system (3.0 mg to 5 mL PBS), which instantly inhibited catalase, as observed by the rapid return to baseline values (Figure 1B,C) [35]. This result concludes that the change of the amperometric signal entirely reflected the enzymatic reaction where O_2_ was produced by catalase.

(3) $$\frac{d\left[O_{2}\right]}{dt}=\frac{1}{2}\times v_{0},$$

(4) $$U=v_{0}\times \alpha .$$

In Equation (3), $d[O_{2}]/dt$ (mM/s) is the rate of oxygen production, which is used to define the initial reaction velocity $v_{0}$ (mM/s) of H_2_O_2_ consumption by the catalase reaction, taking into account that one O_2_ molecule is produced from two H_2_O_2_ molecules, see Equation (1). In Equation (4), α is a conversion factor equal to 300 (L × s/min), going from units of mM/s to units of µmol/min and taking into account the volume of the electrochemical reaction vessel (i.e., 5 mL).

### 2.9. Determination of Apparent Kinetic Parameters of the Catalase Reaction

The apparent Michaelis constant $K_{M}$ (mM) and the apparent maximal reaction velocity $v_{\max}$ (µM/s) for the substrate H_2_O_2_ were determined with the Lineweaver–Burk method, according to Equations (5) and (6), where $v_{0}$ has the same meaning as in Equations (3) and (4), but in units of µM/s. Here, it should be noted that the kinetic constants for catalases must be labeled as “apparent”, due to the fact that catalases do not exhibit Michaelis–Menten kinetics over the complete range of substrate concentration and due to the two-step nature of the catalytic reaction [20]. However, at H_2_O_2_ concentrations below 200 mM, most catalases do exhibit a Michaelis–Menten-like dependence of velocity as a function of substrate concentration [20], as shown in this work.

(5) $$v_{0}=\;\frac{v_{\max}\times \left[H_{2}O_{2}\right]}{K_{M}+\left[H_{2}O_{2}\right]}\;,$$

(6) $$\frac{1}{v_{0}}=\;\frac{K_{M}}{v_{\max}}\times \frac{1}{\left[H_{2}O_{2}\right]}+\frac{1}{v_{\max}}.$$

**Figure 2:**
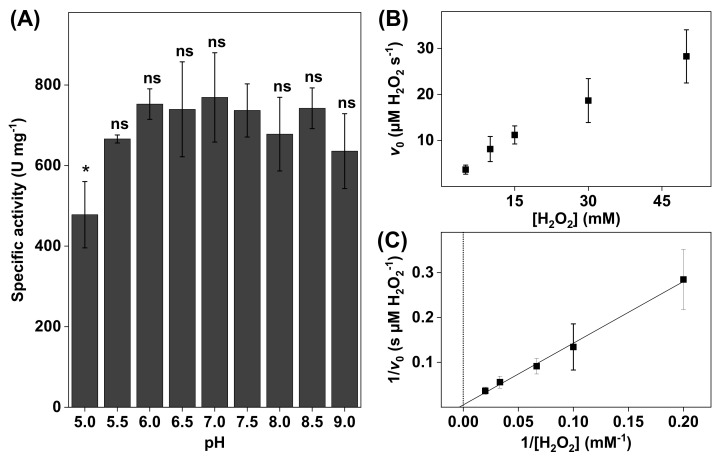
(**A**) The effect of pH on bovine liver catalase activity. All experiments were performed in triplicates (*n* = 3, mean ± SD) with 10 mM H_2_O_2_ and the addition of 3.0 µg catalase. (**B**) Representative data obtained for 3.0 µg of bovine liver catalase in 5 mL of PBS at pH 7.5 presented in the form of a Michaelis–Menten plot where the initial reaction velocities were plotted as a function of H_2_O_2_ concentration (i.e., 5, 10, 15, 30, and 50 mM). (**C**) Lineweaver–Burk plot (r^2^ = 0.996) of the data corresponding to (**B**) where linear extrapolations were used to determine the apparent Michaelis constant ($K_{M}$) and the apparent maximal reaction velocity ($v_{\max}$) according to Equation (6). * *p* < 0.05 and ns = *p* > 0.05.

In these measurements, dispersions of the various biological samples were prepared in PBS to obtain 3.0 µg of neat catalase, 1.0 mg of pulverized DAB or SC, or 2 × 10^5^ keratinocytes per 5 mL of reaction vessel medium (i.e., PBS). Next, the substrate was added to the system to obtain the specified H_2_O_2_ concentration, which ranged between 5 and 50 mM. The initial reaction velocity $v_{0}$ of the O_2_ production was recorded after each H_2_O_2_ addition (Figure 2B). The reaction vessel was emptied and cleaned before performing the next measurement, each time with a fresh dispersion containing the biological sample under investigation. All measurements were repeated three times for each substrate concentration. The reciprocal values of $v_{0}$ were plotted against the reciprocal values of substrate concentrations to obtain Lineweaver–Burk plots (Figure 2C).

### 2.10. Statistics

Statistical comparisons were performed by one-way ANOVA tests using the software OriginPro 2021b. For measurements of catalase activity in dispersions of DAB, SC, or keratinocytes, the ANOVA test was followed by a post-hoc Tukey test. The significance levels were established at * *p* < 0.05, ** *p* < 0.01, and *** *p* < 0.001, while the not significant (ns) level was determined at *p* > 0.05. All experimental data used for statistical analysis are reported as mean ± standard deviation from at least three replicates.

## 3. Results

The overall aim of this study was to employ an oxygen electrode-based method to characterize the catalase activity in biological systems with varying degrees of complexity.

As an initial means of control of the accuracy of the method, the specific catalase activity in units of U/mg was determined according to Equations (3) and (4), where one unit of catalase is defined as the amount of catalase that decomposes 1 µmol of H_2_O_2_ in 1 min at room temperature (22 °C). For this, 3.0 µg of pure catalase was placed in 5 mL of buffer solution (pH 7.5), after which H_2_O_2_ was added to obtain a concentration of 50 mM. Based on these measurements, the specific activity of catalase was determined to be 3600 ± 200 U/mg, which is in agreement with the specified range of 2000–5000 U/mg. However, due to the fact that 50 mM of H_2_O_2_ is a relatively high concentration that may lead to the formation of gaseous O_2_, which was observed in some measurements with high substrate concentrations, it was decided to perform future experiments with a lower concentration of H_2_O_2_, i.e., 10 mM.

In the following, we start by investigating the effect of pH on the catalase activity of neat catalase from bovine liver. Next, we explore the catalase activity in keratinocytes of the cell line HaCaT and explore the effect of different treatments of the cell dispersions before measurements. Following this, we investigate the catalase activity in the skin barrier with a particular focus on stratum corneum (SC) samples with different amounts of melanin (i.e., skin colors). Finally, we evaluate the potential for employing defatted algae biomass (DAB) as a source of the antioxidative enzyme catalase in topical skin care formulations.

### 3.1. Electrochemical Chronoamperometric Measurements of Catalase Activity

To investigate the effect of pH on the enzyme activity, the catalase activity was measured in PBS with pH ranging between 5.0 and 9.0 (see Figure 2A). Notably, the specific activity levels presented in Figure 2A were lower as compared to the results obtained with the higher substrate concentration of 50 mM H_2_O_2_ (i.e., 3600 ± 200 U/mg). However, as beforementioned, to avoid the formation of gaseous O_2_, it was decided to perform all comparative evaluations of the catalase activity with a lower concentration of 10 mM H_2_O_2_.

The results in Figure 2A show that the catalase activity was relatively constant between pH 5.5 and 9.0. The highest activity was observed at pH 7.0, while the lowest activity was recorded at the most acidic pH of 5.0. In addition, the most alkaline solution (i.e., pH 9.0) resulted in a slightly lower catalase activity. In addition, the kinetic parameters were determined at pH 7.5 by linear extrapolation according to Lineweaver–Burk plots (see Figure 2B). From this evaluation, the apparent Michaelis constant and the apparent maximal reaction velocity were determined to be $K_{M}$ = 110 ± 55 mM H_2_O_2_ and $v_{\max}$ = 87 ± 30 µM H_2_O_2_/s, respectively (see Table 1). The reason for this kinetic analysis was to enable comparisons of the kinetic parameters of neat catalase with catalase in various biological systems (vide infra).

### 3.2. Catalase Activity and Enzyme Kinetics in Keratinocytes

**Figure 3:**
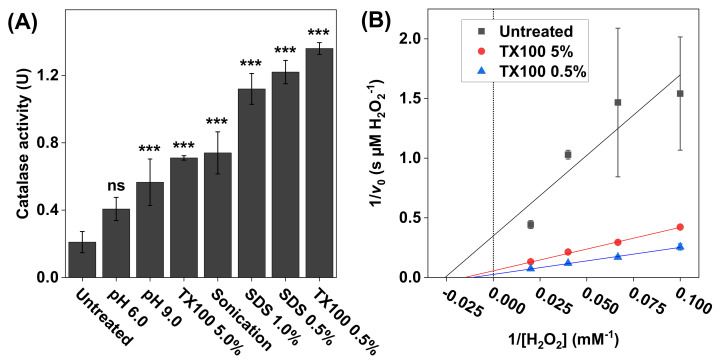
(**A**) The effect of treatment protocol on the catalase activity measured with 2 × 10^5^ HaCaT keratinocytes dispersed in PBS. The treatments included adjusted pH values (6.0 and 9.0), 60s cell incubation in Triton X100 (0.5 wt% or 5.0 wt%) or in SDS (0.5 wt% or 1 wt%), or sonication. All experiments were performed in triplicates (*n* = 3, mean ± SD) with 10 mM H_2_O_2_ and pH 7.5 (if not stated otherwise). (**B**) Lineweaver–Burk plots of representative data obtained with 10, 15, 25, and 50 mM of H_2_O_2_, where linear extrapolations were used to determine the Michaelis constant $K_{M}$ and maximal reaction velocity $v_{\max}$ according to Equation (6). The correlation coefficients were r^2^ = 0.846 for untreated keratinocytes, r^2^ = 0.996 after treatment with 5.0 wt% Triton X100, and r^2^ = 0.997 after treatment with 0.5 wt% Triton X100. *** *p* < 0.001 and ns = *p* > 0.05.

An important mechanism leading to skin disorders is oxidative stress originating in the viable basal layers of the epidermis where the dominating cell type is keratinocytes [4]. The activation of various harmful cell signaling pathways in the epidermis can be triggered by both external (e.g., UV light exposure or xenobiotics) and internal factors (e.g., down-regulation of antioxidative enzymes), resulting in an overall imbalance between antioxidants and oxidants and destructive oxidative stress. To enable simple studies of catalase function in viable and lysed keratinocytes, we employed the present method in a series of experiments. First, the catalase activity of intact HaCaT keratinocyte samples was measured by dispersing 2 × 10^5^ cells in PBS (pH 7.5). The results of this experiment are presented Figure 3A (referred to as untreated). For comparison, the same number of cells was used, but they were treated using various protocols intended to disrupt the cell membranes and release catalase from the intracellular compartments. These protocols included measurements in alkaline (pH 9.0) and acidic (pH 6.0) environments, treatment with SDS or Triton X100 surfactants, and treatment by mild sonication (Figure 3A).

The results in Figure 3A show that the lowest catalase activity was recorded for the untreated cells, which implies that the cell membrane remained intact and thereby limited the diffusion of H_2_O_2_ and/or O_2_ across the cell membrane. In contrast, the results obtained after the various treatment protocols imply that, by disrupting the cell membrane, the transport of both the substrate and the product (i.e., O_2_) was increased, which effectively resulted in elevated catalase activities. In particular, the highest activity was observed after treating the cells with 0.5 wt% Triton X100. Based on these findings, it was decided to explore the catalase kinetics from three selected samples, namely the intact keratinocytes (untreated sample) as well as the samples treated with 0.5 and 5.0 wt% Triton X100 (Figure 3B). In brief, after treatment, aliquots of these samples were added to the reaction vessel containing 10, 15, 25, or 50 mM H_2_O_2_ (Figure 3B). The calculated values of $K_{M}$ and $v_{\max}$ are summarized in Table 1. The highest values of $K_{M}$ (79 ± 22 mM H_2_O_2_) and $v_{\max}$ (36 ± 9.0 µM H_2_O_2_/s) were recorded for the sample treated with 0.5 wt% Triton X100.

### 3.3. Catalase Activity and Enzyme Kinetics in Stratum Corneum (SC)

**Figure 4:**
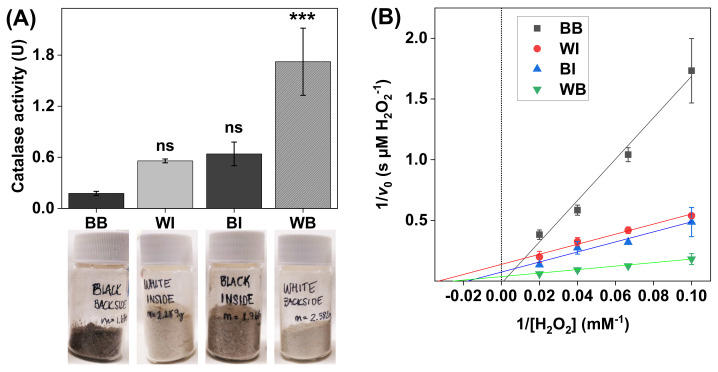
(**A**) Catalase activity in SC samples with noticeable differences in skin color (i.e., melanin content). All experiments were performed in triplicates (*n* = 3, mean ± SD) with 10 mM H_2_O_2_ at pH 7.5 and with the addition of 1.0 mg of SC sample in 5 mL of PBS. (**B**) Lineweaver–Burk plots obtained with 10, 15, 25, and 50 mM of H_2_O_2_, where linear extrapolations were used to determine the Michaelis constant $K_{M}$ and maximal reaction velocity $v_{\max}$ according to Equation (6). The correlation coefficients were r^2^ = 0.986 for black backside (BB), r^2^ = 0.980 for white inside (WI), r^2^ = 0.980 for black backside (BI), and r^2^ = 0.999 for white backside (WB). *** *p* < 0.001 and ns = *p* > 0.05.

The outermost skin layer represents the main skin barrier and this layer is usually referred to as the stratum corneum (SC). The SC represents a thin membrane (ca. 20 µm) that is composed of corneocytes (dead cells), which are embedded in an extracellular lipid lamellae [36]. The major fraction of both the keratin filaments inside the corneocytes and the extracellular lipids are solid-like [16,17,37,38]. In several aspects, the SC membrane is thought of as non-viable tissue. However, the SC represents a dynamic structure in which several enzymes are embedded in a functional state, such as enzymes of the antioxidative system of the skin. For example, the enzyme superoxide dismutase converts harmful ROS to the less harmful ROS H_2_O_2_, which in turn is converted by catalase to harmless H_2_O and O_2_. The body’s antioxidative system also includes low molecular weight molecules, such as melanin, that absorb and scatter incoming UV radiation without causing harm, thereby preventing the formation of oxygen radicals [39,40]. Despite this comprehensive antioxidative system, our body remains susceptible to oxidative damage from harmful ROS and exposure to UV light. With this as background, the aim here was to employ the present methodology to investigate the catalase activity and enzyme kinetics in SC samples with clear variations of the skin color, indicating different amounts of melanin. For this, four types of SC sample batches, obtained from either the inside or the backside of darkly or lightly pigmented pig ears, were investigated (see Figure 4A).

The results in Figure 4A show clear catalase activity variations in the SC samples. In particular, the highest catalase activity was recorded for the SC samples originating from the backside of pale ears (i.e., WB). Interestingly, the catalase activity of the SC samples from the same batch of ears, but taken from the inside (i.e., WI), was approximately three times lower. Further, the opposite relationship was recorded for the SC samples collected from the darker tissue, where the catalase activity was approximately 3–4 times lower in the samples originating from the backside of the ears (i.e., BB), as compared to the samples from the inside (i.e., BI).

The results from the Lineweaver–Burk plots, corresponding to the different SC samples, are presented in Figure 4B, while the calculated apparent Michaelis constants ($K_{M}$) and apparent maximal reaction velocities ($v_{\max}$) are summarized in Table 1. In brief, the data corresponding to the WI, BI, and WB samples showed relatively similar values of $K_{M}$, ranging between 30 ± 13 and 51 ± 45 mM H_2_O_2_, while the average $v_{\max}$ value for the WB samples (34 ± 27 µM H_2_O_2_/s) was notably higher as compared to the data obtained from the WI and BI samples (i.e., 7.3 ± 2.9 and 9.3 ± 3.3 H_2_O_2_/s, respectively). Here, it should also be pointed out that the linear extrapolation failed to yield a positive value of the intercept for the data corresponding to the BB samples, which prevented the analysis of the kinetic parameters in this case.

### 3.4. Catalase Activity and Enzyme Kinetics in Defatted Algae Biomass (DAB)

**Figure 5:**
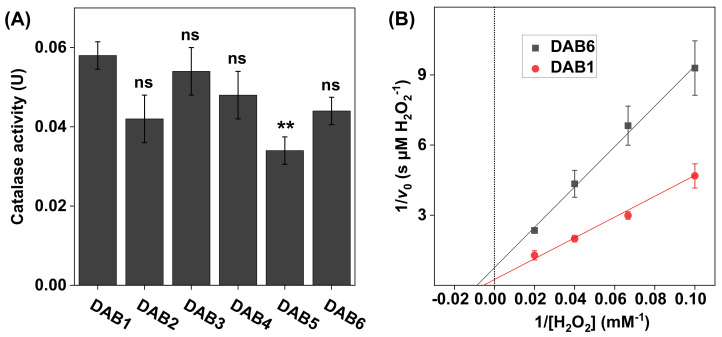
(**A**) Catalase activity in DAB samples harvested at different time points between 2016 (DAB1) and 2018 (DAB6). All experiments were performed in triplicates (*n* = 3, mean ± SD) with 10 mM H_2_O_2_ at pH 7.5 and 1.0 mg of DAB sample in 5 mL of PBS. (**B**) Lineweaver–Burk plots for DAB1 and DAB6 obtained with 10, 15, 25, and 50 mM of H_2_O_2_, where linear extrapolations were used to determine the Michaelis constant $K_{M}$ and maximal reaction velocity $v_{\max}$ according to Equation (6). The correlation coefficients were r^2^ = 0.993 for DAB1 and r^2^ = 0.996 for DAB6. ** *p* < 0.01 and ns = *p* > 0.05.

One of the main goals of many skin care products is to neutralize ROS overproduction and thereby delay the onset of skin aging by slowing down any processes that lead to structural and visual skin aging effects over time. Considering that catalase is recognized as one of the most important antioxidative enzymes to combat ROS [21], we investigated the potential of using defatted algae biomass (DAB) as a source of catalase for skin care formulations. The DAB represents a byproduct derived from the diatom microalga *Phaeodactylum tricornutum* after lipid extraction. In brief, *Phaeodactylum tricornutum* is a primary producer of the omega-3 fatty acid EPA (eicosapentaenoic acid) and is grown primarily to obtain an oil rich in omega-3 fatty acids as a food supplement. However, during this manufacturing process, the DAB fraction remains as a potential source of various beneficial chemical constituents to be employed, for example, in skin care products. Thus, as an initial examination of this possibility, we investigated if DAB may represent a suitable source of the antioxidative enzyme catalase. To determine the catalase activity in six different batches of DAB, the oxygen electrode-based method was employed. The samples were numbered chronologically from the earliest acquired (i.e., DAB1 harvested in 2016) to the latest (i.e., DAB6 harvested in 2018) (see Figure 5A).

The results in Figure 5A show that, overall, the catalase activity of the DAB samples was similar in all samples. Further, the apparent kinetic parameters, calculated based on the results in Figure 5B, were also similar for both samples with $K_{M}$ values determined to be 130 ± 33 mM H_2_O_2_ for DAB1 and 110 ± 71 mM H_2_O_2_ for DAB6, while the corresponding values of $v_{\max}$ were 3.0 ± 0.71 µM H_2_O_2_/s and 1.3 ± 0.78 µM H_2_O_2_/s, respectively (see Table 1).

### 3.5. The Effect of pH on Catalase Activity in Stratum Corneum (SC) and Defatted Algae Biomass (DAB) Samples

**Figure 6:**
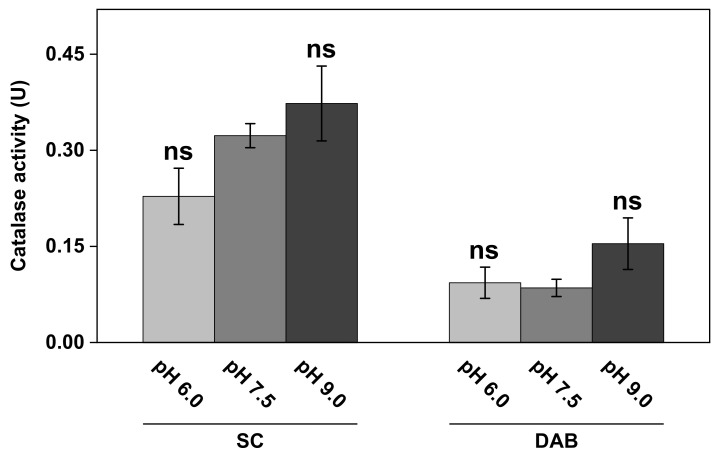
The effect of pH on catalase activity in SC and DAB samples. All experiments were performed in triplicates (*n* = 3, mean ± SD) with 10 mM H_2_O_2_ and 1.0 mg of SC or DAB samples in 5 mL of PBS. ns = *p* > 0.05.

To employ catalase as an antioxidative skin care ingredient, it is necessary to develop a skin care formulation that provides appropriate conditions for the enzyme to maintain its functionality and activity. In relation to this, the pH of the potential skin care formulation is important to consider, which will ultimately depend on the specific chemical composition of the formulation. Further, it is expected that the pH of the aqueous microenvironment of the skin tissue can vary between 4.5 and 6.0 [41,42,43]. In particular, in the case of a defective skin barrier, such as an open wound, the pH is normally significantly alkaline with values as high as 9.0 [44]. Thus, it is clear that pH fluctuations are expected in the case of a realistic topical skin care application. Considering this, it is relevant to investigate the influence of the pH on the catalase activity, both of SC samples as such, and the DAB as a potential catalase source. Therefore, the oxygen electrode-based method was employed to determine the enzyme activity in both SC and DAB samples at different pH values (see Figure 6).

The results in Figure 6 show that the catalase activity in the complex biological samples of SC and DAB was dependent on the pH of the medium to some extent. In particular, higher activity was observed at the most alkaline pH value (i.e., pH 9.0) for both the SC as well as the DAB samples. Here, it should be noted that the SC samples, corresponding to the results in Figure 6, were obtained from the same batch as the WI presented in Figure 4 (i.e., inside of white ears). However, this batch of samples was stored in vacuum for several weeks, which can explain the lower catalase activity observed in Figure 6, as compared to the results from this batch in Figure 4. Further, the apparent kinetic parameters at pH 7.5 of these samples, after prolonged storage in vacuum, were determined to be $K_{M}$ = 140 ± 67 mM H_2_O_2_ and $v_{\max}$ = 15 ± 6.5 µM H_2_O_2/_s, which should be compared to the values of 30 ± 13 mM H_2_O_2_ and 7.3 ± 2.9 µM H_2_O_2_/s, respectively, for the fresher samples (see WI in Table 1).

## 4. Discussion

### 4.1. The Effect of pH on Catalase Activity in Biological Samples

In the first set of experiments, the activity of neat bovine liver catalase was evaluated in PBS with pH values adjusted between 5.0 and 9.0 (Figure 2A). In conclusion, based on the results from triplicate measurements, the enzyme activity was observed to remain at an overall stable level at pH values varying between 6.0 and 7.5, while a noticeable decrease of approximately 40% was observed at pH 5.0 (Figure 2A). Further, the activity at alkaline pH values was also slightly lower, as compared to the activity at pH 7.0, with approximate decreases of 10% at pH 8.0 and 20% at pH 9.0 (Figure 2A). However, at pH 8.5, the corresponding decrease was only around 5%. Overall, this bell-shaped pH-activity curve, centered around pH 7.0, is in line with previous studies showing a decrease in the activity of native catalase at pH 4.5–5.0 of about 20–80% and an approximately 10–60% decrease at pH 8.0–8.5 [45,46,47]. On the other hand, a study by Costa et al. showed that catalase retained more or less constant activity between pH 7 and 10, while a significant decrease was observed at higher pH values [48]. Possibly, the time of catalase exposure to the acidic or alkaline medium, which in this study was no longer than 15 min, could be the reason for this kind of discrepancy.

Taken together, the results presented in Figure 2A confirm the well-established bell-shaped pH-activity curve of neat catalase, centered around pH 7.0 [45,46,47]. This is an important finding, which implies that any observations of, for example, increased catalase activity at moderately acidic (i.e., pH 6.0) or alkaline (i.e., pH 9.0) conditions are likely related to effects resulting in higher substrate accessibility to catalase in the investigated biological sample and/or elevated diffusion of O_2_ from the catalase site to the oxygen electrode and not due to higher catalase activity per se. Considering this, it is more appropriate to classify any changes of the catalase activity in terms of effective catalase activity in the following discussion.

Interestingly, the keratinocytes exposed to solutions with pH 6.0 and pH 9.0 resulted in elevated effective catalase activity levels (see Figure 3A). In particular, the alkaline treatment resulted in a notable increase in the effective catalase activity, as compared to pH 7.5. These effects were most likely caused by an altered charge status of the phospholipid headgroups and subsequent electrostatic repulsion, leading to defects in the lipid bilayer and increased cell membrane permeability of both H_2_O_2_ and O_2_ into and out of the cytosol [49]. The corresponding pH effect on the effective catalase activity was different for the SC and DAB samples (see Figure 6). In this case, only the alkaline treatment resulted in elevated effective catalase activity. Starting with the results obtained with the SC samples, the natural pH of the skin barrier oscillated around 4.5–5.0, where the acidic mantle primarily protected against antimicrobial invasion and provided healthy conditions for the resident skin microflora [50]. Further, it is well-known that the skin pH is an important factor for maintaining the integrity of the skin barrier. For example, it was shown by electrical impedance studies that treatment of the SC tissue for 24 h in a solution with pH 5.5 improves skin integrity, as judged from the observed increase in the skin membrane’s electrical resistance [51]. In contrast, the same treatment of the skin membrane in solutions with pH 7.4 and 8.8 resulted in a rather drastic decrease in the skin barrier’s resistance [51]. On a molecular level, this can be explained in a similar manner as given above in connection to the keratinocytes. In other words, treatment in alkaline solutions modifies the charge status of the molecular components of the SC tissue, such as the fatty acids residing in the extracellular lipid matrix or titratable amino acid residues of the keratin filaments, which ultimately leads to swelling induced by electrostatic repulsion forces, loss of tissue integrity, and increased permeability characteristics [51]. In conclusion, the observed elevated effective catalase activity in the SC samples, after treatment in solution with increasing alkalinity, can therefore be explained by loss of SC tissue integrity, leading to improved substrate accessibility for catalase and overall increased transport characteristics of both H_2_O_2_ and O_2_ into and out of the SC tissue.

A similar explanation can most likely be assigned to explain the notable higher effective activity of catalase in the DAB sample, which is composed mainly of proteins and carbohydrates with several functional groups that are susceptible to alterations in charge status depending on the specific pH [52]. However, in the case for DAB, the pH effect, leading to increased effective catalase activity, was only observed at pH 9.0, while pH 6.0 and 7.5 resulted in similar results (see Figure 6).

### 4.2. Catalase Activity in Intact and Lysed HaCaT Keratinocytes

To enable investigations of the effect of oxidative stress, such as exposure to UV radiation or exposure towards various xenobiotics, on the catalase expression and function of keratinocytes, it is crucial to optimize the experimental protocol employed before measuring the catalase activity with the oxygen electrode. Therefore, the presented method was employed to investigate the effect of various treatment protocols aiming to disrupt the keratinocyte cell membranes and thereby increase the accessibility of intracellular catalase to the substrate, as well as increasing the diffusion of O_2_ to the oxygen electrode, without inactivation of the catalase function (see Figure 3). For this, mild sonication was tested and compared with two surfactants, Triton X100 (0.5 wt% and 5.0 wt%) and SDS (0.5 wt% and 1 wt%). In brief, mild sonication resulted in an approximately 3.5-fold increase in the effective catalase activity, as compared to the untreated cells. Evidently, more exhaustive sonication could potentially be employed to disrupt the cell membrane to a greater extent and thereby increase the accessibility of intracellular catalase. However, this was not investigated due to the fact that extensive sonication is known to cause denaturation of the enzyme [53]. In conclusion, treatment with mild sonication represents a simple procedure with relatively good outcomes in terms of effective catalase activity.

Between all tested treatments, incubation of keratinocytes in 0.5% Triton X100 was most effective, showing an approximately 7-fold increase in the effective catalase activity, as compared to the untreated cells. Interestingly, incubation in 5% Triton X100 resulted in a drastically lower effective catalase activity, as compared to the lower concentration of the same surfactant (approximately two times lower). This implies that this non-ionic surfactant has a tendency to disrupt the tertiary structure of catalase by breaking the native protein intermolecular interactions, leading to the denaturation of catalase at high concentrations [54]. Likewise, a similar concentration dependance was observed for the anionic detergent SDS, which is a well-known agent for lysing cell membranes as well as denaturing proteins [54]. Considering that the anionic SDS substance is expected to be more efficient in deactivating catalase, by denaturing its tertiary structure (as compared to Triton X100, 647), 1% was chosen as the highest concentration for incubation of the keratinocytes. Analogously to the situation of Triton X100, the cells incubated in the highest concentration of SDS (i.e., 1%) showed slightly lower effective catalase activity, as compared to incubation in 0.5% SDS. Taken together, these findings suggest that further investigations with lower surfactant concentrations should be conducted to identify the optimal surfactant concentration to be used for enabling efficient cell membrane disruption, at the same time as catalase inhibition is avoided due to denaturation of the enzyme.

### 4.3. Catalase Activity in the Stratum Corneum with Varying Skin Color

The defense system against UV radiation (UVR) of the skin organ depends on several factors. In particular, melanin serves as a key protective agent against damaging effects of UV light, which can be illustrated by the inverse correlation between the melanin content of human skin and the incidence of skin carcinomas and melanomas induced by UVR [40]. Two main types of melanin are recognized, namely eumelanin, which is associated with darkly pigmented skin, and pheomelanin, which is mainly present in bright skin [40,55]. Eumelanin acts as a UV filter and possesses scavenger properties towards free radicals induced by UVR, while pheomelanin is less effective as a UV filter and may even contribute to skin carcinogenesis by producing free radicals in response to UVR [39,40]. Considering this, it is reasonable to hypothesize that parts of skin with light pigmentation that are exposed more to UV light should be associated with a more comprehensive system of antioxidative enzymes, such as catalase, to compensate for a lower antioxidative protection from dark pigment (i.e., eumelanin). In fact, the results in Figure 4A clearly show that the white SC samples were, in general, associated with higher catalase activity, as compared to the darkly pigmented SC samples. Further, the highest catalase activity was observed in the SC samples taken from the backside of white ears, which were more exposed to UVR due to fact that the white pig ears were rather large and folded. Thus, it is reasonable to assume that the backside of the ear should contain more catalase, as compared to the inside of the ear that is protected more against UVR and, therefore, ought to have a lower requisite to express catalase. Hypothetically, this may explain the relatively low catalase activity of SC samples taken from the inside of the same batch of white ears (Figure 4A). In line with this reasoning, a previous study concluded that chronically exposed skin sites of human subjects are associated with higher catalase activity in the epidermis [56]. With respect to the darkly pigmented skin, the opposite relationship was observed with about three times higher catalase activity for the SC samples obtained from the inside of the ears than the sample from the backside of the same batch of ears (see Figure 4A). Potentially, this finding may be explained by the presence of a dense layer of coarse black hair on the backside of the ears, which may act as a protective layer against UVR [55], while the hair density of the inside of the darkly pigmented ears was lower. Further, the darkly pigmented ears were, in general, smaller, as compared to the white ears, which implies that these ears were less folded (i.e., the inside of the ear was exposed more to UVR). Speculatively, this may lead to an overall higher expression of catalase in the skin of the inside of the black ears, while the skin from the backside of the ears is overall protected more from UV light, leading to lower catalase expression.

In summary, the present results imply that skin that is exposed more to UV radiation and/or has lower amounts of melanin, in particular lower amounts of eumelanin of darkly pigmented skin, is associated with higher catalase activity in the SC. Interestingly, this finding is not in agreement with the conclusions of a previous study performed with primary cultures of human melanocytes, showing a positive correlation between melanin content and catalase activity as determined by spectrophotometry of lysed and filtrated cells [39]. In other words, darkly pigmented melanocytes possessed higher catalase activity, while lightly pigmented melanocytes showed lower levels of catalase activity [39]. However, since the SC tissue and viable melanocytes represent two completely different biological systems, it is not straightforward to draw any certain conclusions of the meaning of this discrepancy without further investigations. Still, the present results illustrate the potential of employing the oxygen electrode-based method for investigating the catalase activity in SC samples with different skin colors (i.e., melanin contents), as well as keratinocytes, which is promising for further and more detailed studies of various cell types, such as melanocytes.

### 4.4. Defatted Algae Biomass as a Potential Source of Catalase in Skin Care Formulations

In this work, DAB was investigated as a potential source of catalase to be employed in skin care formulations. Even though the SC and DAB represent two completely different biological systems and were also prepared according to quite different protocols before performing the experiments (which may have influenced the observed catalase activity), the results presented in Figure 6 show that the catalase activity in the DAB and SC samples was on a comparable scale. This finding indicates that DAB can potentially be employed in skin care as a source of the antioxidative enzyme catalase. Further, it can be noted that the DAB is expected to contain several other beneficial chemical constituents, in addition to catalase, such as polysaccharides, sulphated polysaccharides, glucosyl glycerols, pigments (e.g., fucoxanthin, scytonemin, phycobiliproteins, carotenoids, etc.), and polyphenols, which, taken together, may act in synergy to enhance beneficial effects and prevent harmful effects, such as oxidative stress and exposure to UV light, in skin care applications.

### 4.5. Comparison of Kinetic Parameters of Catalase in Various Biological Samples

Previous studies on native skin catalase function have focused on the inhibition of catalase exposed to UV light or various commonly administered drug molecules and describe changes in enzyme kinetic parameters [57,58]. However, the methods employed in this kind of study require relatively extensive purification of catalase, which is costly and time-consuming. Therefore, considering the great importance of catalase activity in the epidermis for balancing ROS levels and preventing inflammation, there is an unmet need for fast and reliable methods allowing for direct investigation of the catalase inhibition process and the influence of potential harmful factors, such as air pollutants, cosmetic ingredients, or microbial toxins, on the apparent kinetic parameters of catalase. Here, we used the oxygen electrode-based method to determinate the apparent kinetic parameters $K_{M}$ and $v_{\max}$ of catalase in keratinocytes, SC samples, and DAB samples. The apparent $K_{M}$ represents the affinity of the enzyme to bind the substrate, while the apparent $v_{\max}$ reflects the catalytic rate of catalase under the specified conditions [59].

The results obtained for untreated HaCaT keratinocytes, and the corresponding cells after treatment with the surfactant Triton X100, were considerably different. Intact keratinocytes had the lowest apparent $K_{M}$ and $v_{\max}$, which could be rationalized by the limited permeability of the substrate and the product in and out from the cytosol and across the cell membrane, which effectively give rise to an efficient binding of the substrate but also a low turnover rate. The highest values of the apparent $K_{M}$ and $v_{\max}$ were obtained for cells treated with 0.5% Triton X100, while the cells lysed with 5% Triton X100 had values in between the untreated cells and the lowest surfactant concentration (see Table 1). These findings indicate that Triton X100, although widely used in isolation protocols of various enzymes, may have inhibitory effects on the catalase function, especially at relatively high concentrations. In other words, Triton X100 disturbs the substrate binding, giving rise to higher values of the apparent $K_{M}$, while the effective turnover rate is elevated due to the disruption of the cell membrane and the free diffusion of H_2_O_2_ and O_2_ from the active site to the electrode.

In general, the apparent $K_{M}$ and $v_{\max}$ values, obtained for the SC samples with varying skin color, were found to be very consistent (see Table 1). However, the apparent $v_{\max}$ of catalase in the SC sample obtained from the backside of the white ear was higher as compared to the other samples, which is likely due to a higher amount of catalase in this sample batch. This finding is in line with the observed elevated catalase activity of this SC sample batch, which was discussed above. Likewise, the apparent $K_{M}$ and $v_{\max}$ values determined for the DAB samples were similar overall (see Table 1). However, the apparent $v_{\max}$ value for DAB1 was about twice as high as compared to the corresponding value of DAB6. Potentially, this discrepancy may be explained by variations of the specific conditions employed during cultivation of the microalga *Phaeodactylum tricornutum*, such as the amount of CO_2_ supplied and the level of artificial UV light used, which may have influenced the expression of catalase. Taken together, the results from these experiments illustrate the potential for using the present method in assessing the effect of various treatment protocols or comparing the apparent kinetic parameters of catalase in different biological samples.

## 5. Conclusions

The results presented in this work illustrate the versatility of the oxygen electrode-based method for investigating the function of catalase in biological samples of varying complexity, such as keratinocytes, excised stratum corneum, and defatted algae biomass. In particular, the method consists of a simple set-up (see Figure 1A), relies on highly available instrumentation and reagents, and provides relatively fast measurements (e.g., the enzyme kinetics can be determined from measurements at multiple substrate concentrations in a few hours). By using this method, we demonstrate that defatted algae biomass, a byproduct from food supplement manufacturing, retains quantifiable and hopefully valuable catalase activity after oil extraction and prolonged storage time. The catalase activity of this byproduct is approximately four orders of magnitude lower than that of pure enzyme activity, but is comparable to the catalase activity found in excised pig stratum corneum. Finally, the substrate of catalase, i.e., the ROS H_2_O_2_, has been increasingly recognized as a key molecule for redox signaling with several metabolic and biochemically relevant roles [1,60]. This further enhances the relevance of developing a versatile method to determine the catalase activity in complex and native biological systems in the presence of H_2_O_2_, in combination with complementary studies that aim to investigate how H_2_O_2_ may induce oxidative stress in the same systems.

## Figures and Tables

**Table 1:** Apparent kinetic parameters of neat catalase and catalase in various biological systems, including keratinocytes exposed to different treatment protocols, stratum corneum (SC) samples with visually different melanin contents, and defatted algae biomass (DAB) samples. Linear extrapolations were used to determine the apparent Michaelis constants $K_{M}$ (mM H_2_O_2_) and the apparent maximal reaction velocity $v_{\max}$ (µM H_2_O_2_/s) according to Equation (6). All experiments were performed in triplicates for each concentration of H_2_O_2_ (i.e., at 10, 15, 25, and 50 mM H_2_O_2_) in PBS at pH 7.5.

Sample		Treatment	$K_{M}$	$v_{max}$
Bovine liver catalase (3.0 µg)		Untreated	110 ± 55	87 ± 30
HaCaT keratinocytes (2 ×10^5^ cells)		Untreated	30 ± 12	2.2 ± 0.53
		Triton X100 0.5 wt%	79 ± 22	36 ± 9.0
		Triton X100 5 wt%	56 ± 11	16 ± 2.9
Stratum Corneum (SC, 1.0 mg)	Black Backside (BB)	Sonication	N/A	N/A
	White Inside (WI)	Sonication	30 ± 13	7.3 ± 2.9
	Black Inside (BI)	Sonication	30 ± 11	9.3 ± 3.3
	White Backside (WB)	Sonication	51 ± 45	34 ± 27
Defatted Algae Biomass (DAB, 1.0 mg)	DAB1	Sonication	130 ± 33	3.0 ± 0.71
	DAB6	Sonication	110 ± 71	1.3 ± 0.78

## Data Availability

All data are available from the corresponding author upon request.
